# Supplementary material for: N2B27 media formulations influence gastruloid development
Source: Development. 2025 Nov 25;152(22):dev204774. doi: 10.1242/dev.204774 (PMC12687332; doi:10.1242/dev.204774)
Supplement: Supplementary information [file develop-152-204774-s1.pdf]

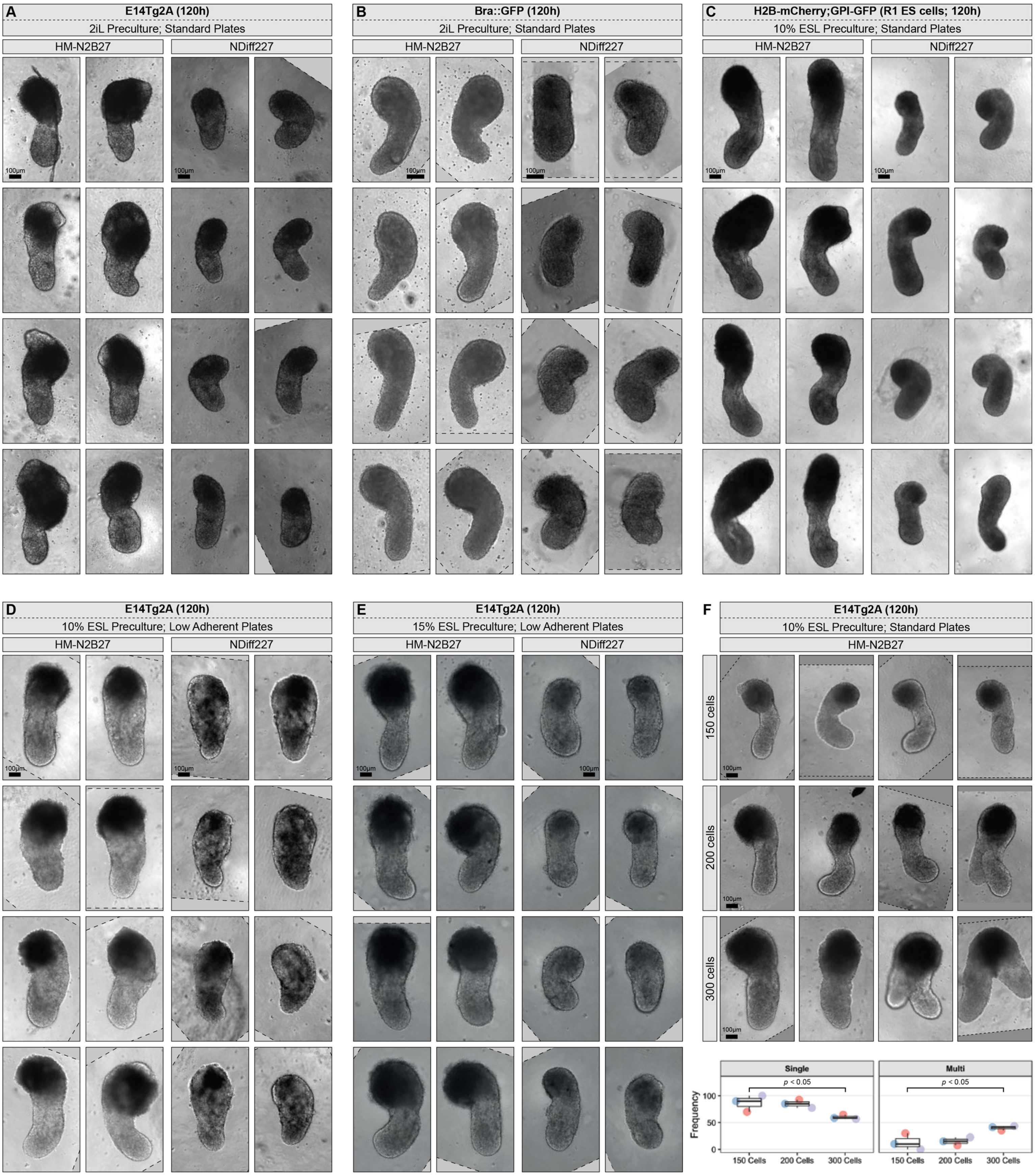

Development • Supplementary information

Fig. S1. HM-N2B27 and NDiff227 gastruloids show consistent phenotypic differences across cell types and culture conditions. (**A-E**) Example images of 120h single axis gastruloids generated from different ES cell lines and genetic backgrounds using different batches of both NDiff227 and HM-N2B27, different pre-culture conditions (e.g. 2iL, 15% ESL) and different gastruloid plates (standard or low-adherent). Overall, HM-N2B27 consistently produce larger and longer gastruloids than NDiff227, regardless of the pre-culture conditions or the 96-well plates used. Images are representative of at least three independent biological replicate experiments, each containing between 40 and 70 individual gastruloids. (**F**) Representative images of single and multi axis 120h HM-N2B27 gastruloids (10% ESL pre-culture) developed using different cell seeding numbers. A quantitative analysis is shown using 3 biological replicate population averages for the frequency of single or multi axis gastruloids (pooled gastruloids, per experiment). The total number of gastruloids in each replicate experiment is between 150 and 300 individual gastruloids. Statistical significance was determined through a 1-way ANOVA followed by Tukey's post-hoc test.

Development • Supplementary information

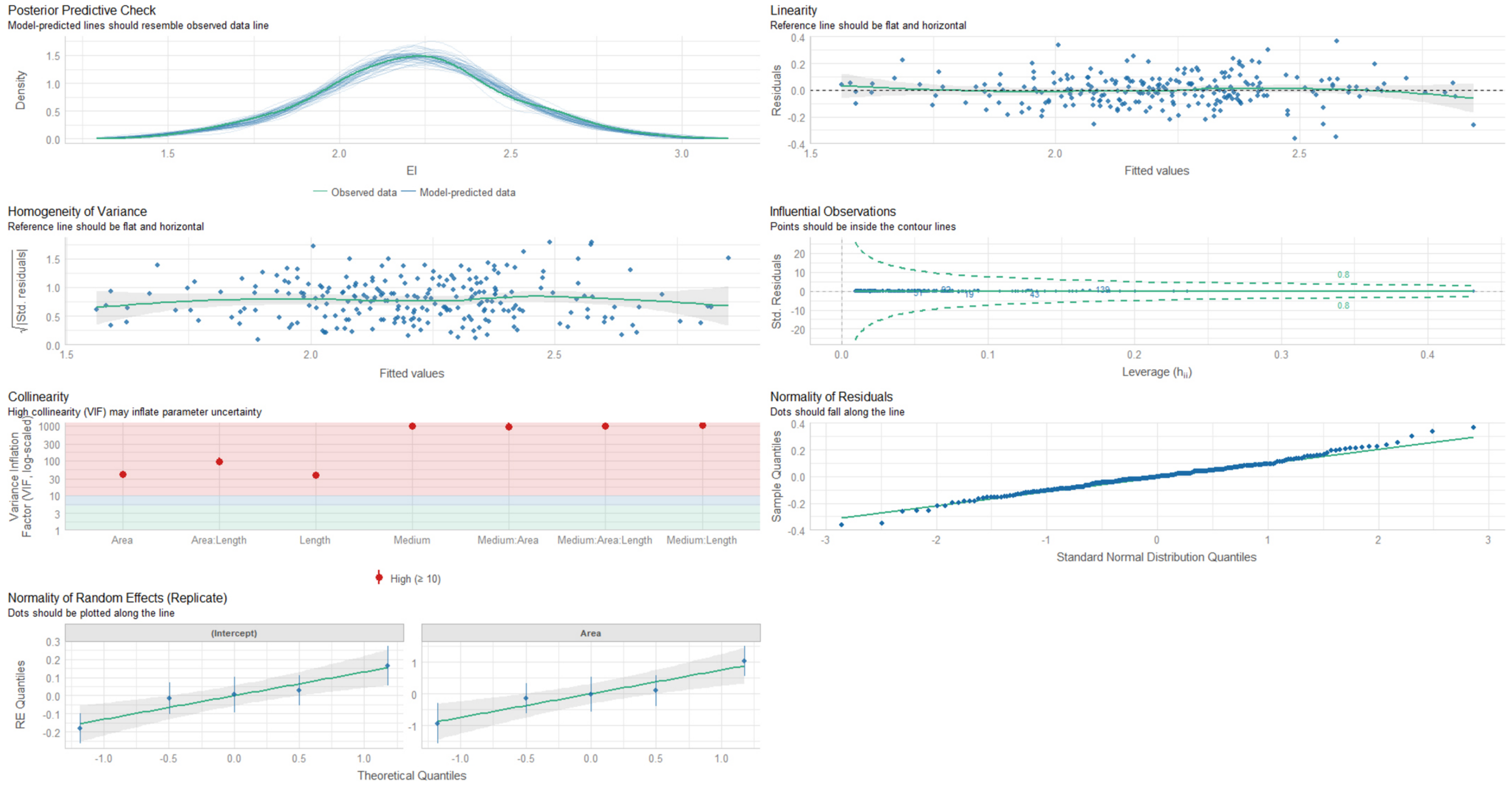

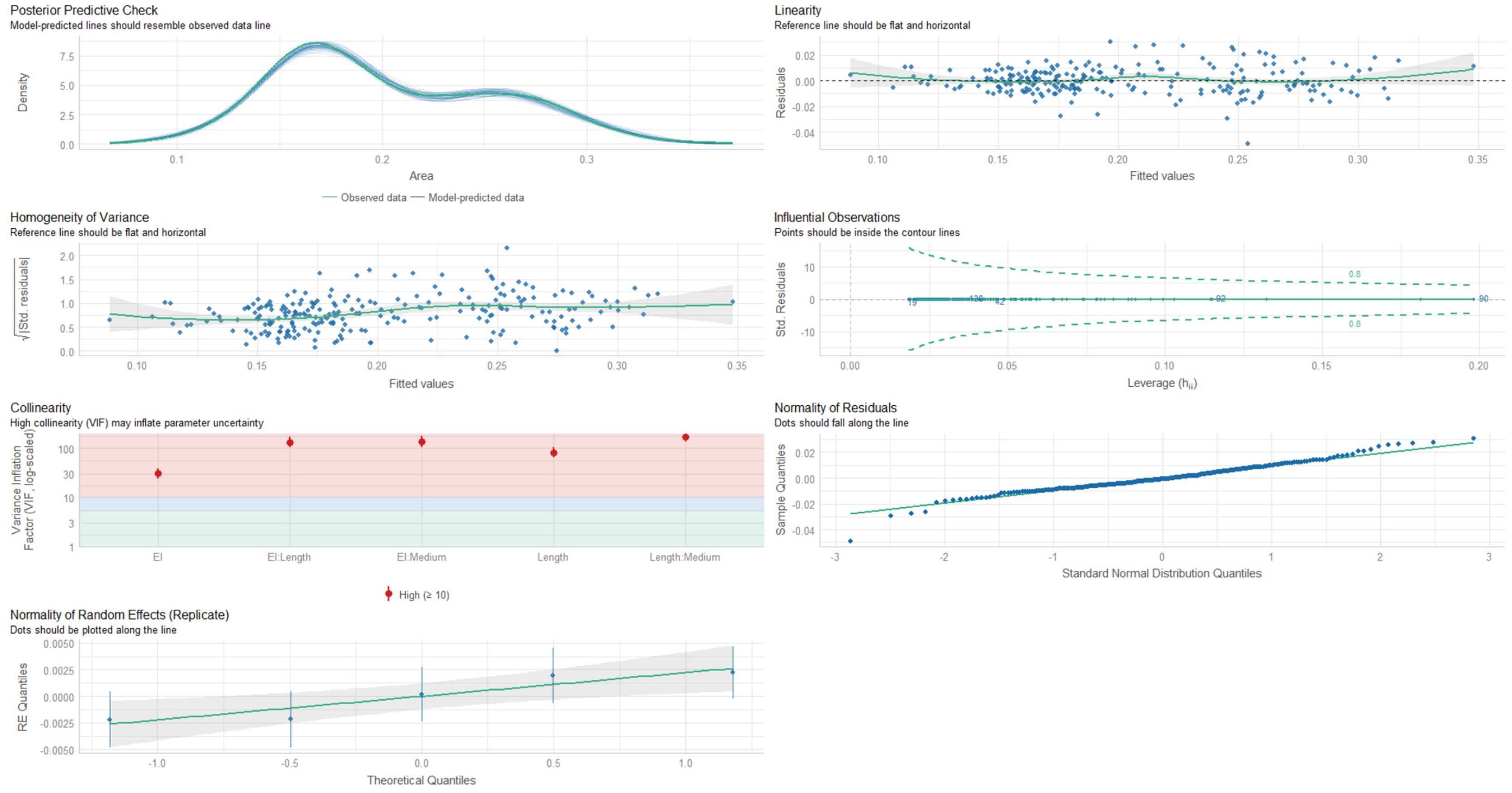

Development • Supplementary information

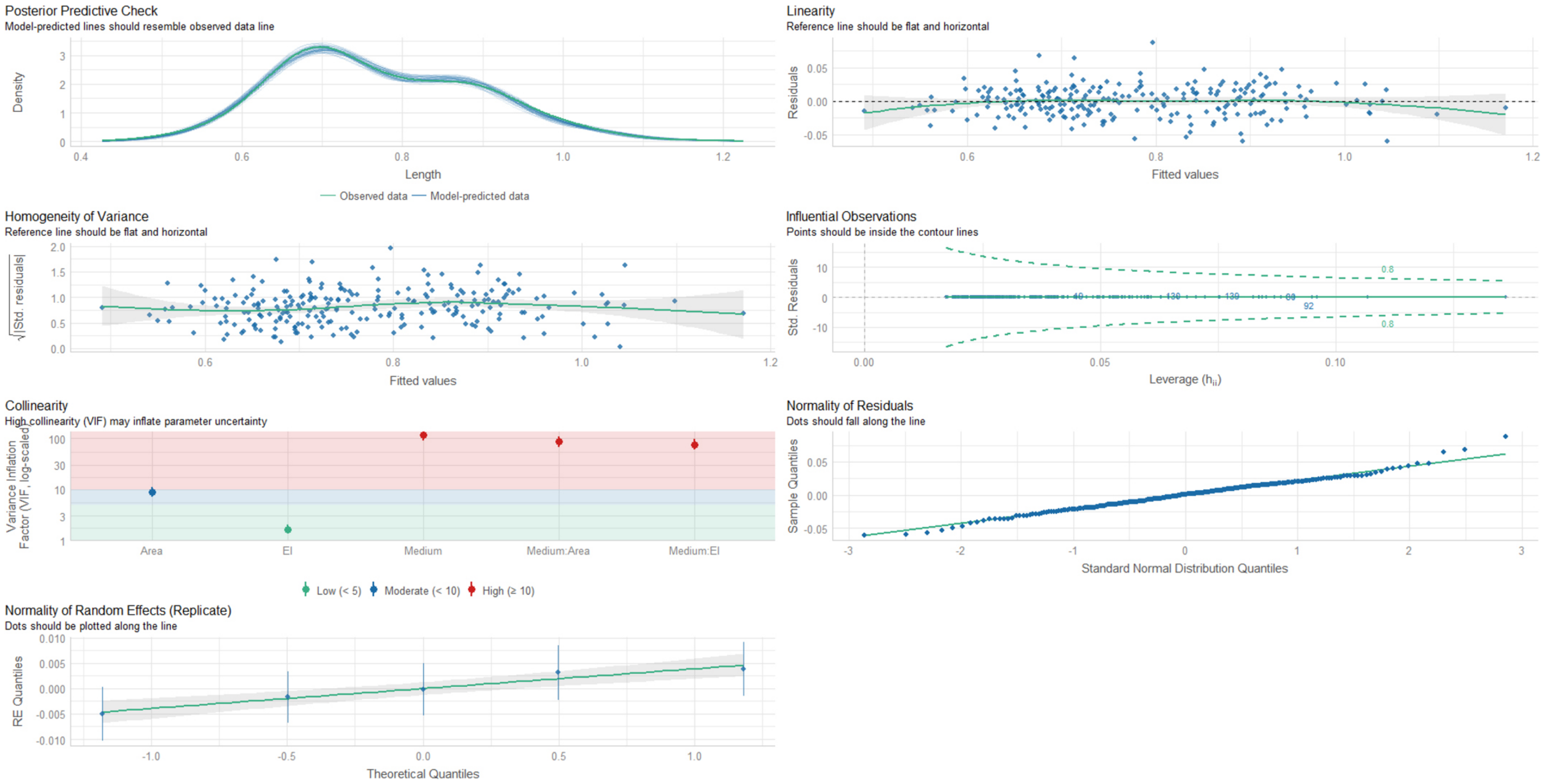

Fig. S2. Performance analysis of mixed effects a model to validate statistical conclusions made for elongation index (A), area (B) and length (C) of gastruloids in Fig. 1C. The Mixed-effects model used was as follows “lmer(EI ~ MediumAreaLength + (Area|Replicate)) “, “lmer(Area ~ EI*Length + Medium:EI + Medium:Length + (1|Replicate))“ and “lmer(Area ~ EI*Length + Medium:EI + Medium:Length + (1|Replicate))“ for elongation index, area and length, respectively. Models were chosen based on AIC and statistical assumptions including homoscedasticity and normal distribution of residuals. Fixed variable interactions were dropped when non- significant. Predictive data was generated using the “predictInterval” function with 1000 simulations to generate overlaying box plots with 95% confidence intervals found in **Fig. 1C2**. Libraries used: car, data.table, emmeans, lme4, merTools, readxl, performance.

Development • Supplementary information

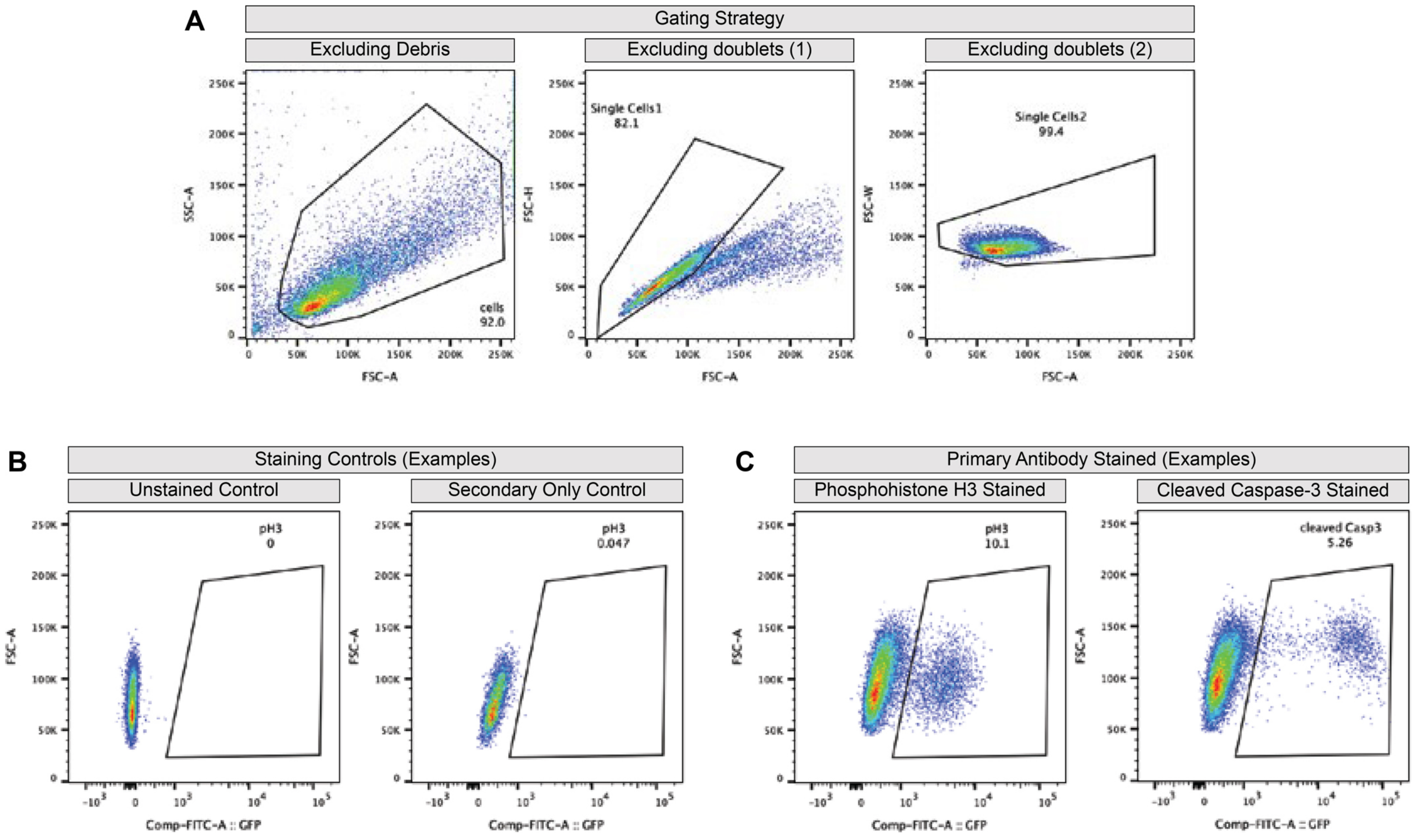

Fig. S3. Gating strategy for flow cytometry. A representative example of fixed and stained cells to illustrate the gating strategy for the data shown in Fig. 1F. (**A**) Forward scatter area (FSC-A) and side scatter area (SSC-A) discriminated cells from debris (left), with the forward scatter pulse amplitude (FSC-H; middle) and width (FSC- W; right) used to exclude doublets and only select single cells. (**B**) Unstained cells (left) and cells stained with only the secondary antibody (alexa-488) established the baseline for negative cells. (**C**) Examples of cells that were positive for either phospho-histone H3 (left) or cleaved caspase-3 (right).

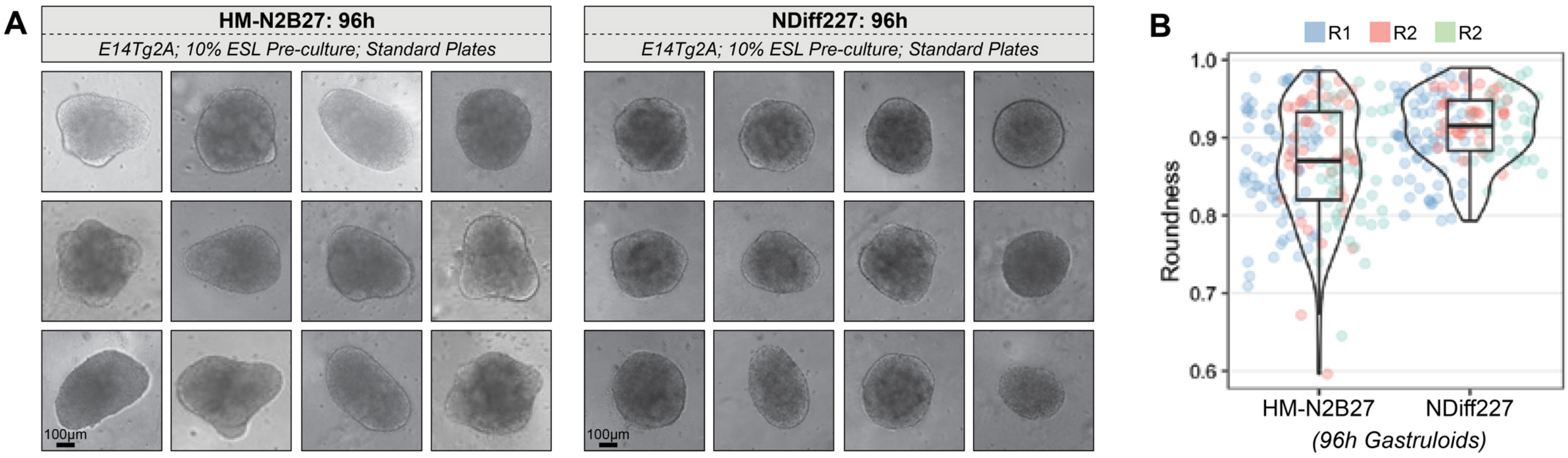

Fig. S4. HM-N2B27 and NDiff227 gastruloids exhibit a distinct morphology at 90h and 96h. (**A**) Example images from several independent experiments of E14Tg2A gastruloids cultured with either HM-N2B27 or NDiff227 media. In contrast to NDiff227 gastruloids that mostly display a round morphology, the majority of HM-N2B27 gastruloids had already started the elongation process at 96h. Scale bar indicates 100 µm. The rightmost image of the middle row of HM-N2B27 gastruloids is from Fig. 2. (**B**) Quantitative analysis of the roundness ($Perimeter^2/4\pi$ x Area) of individual gastruloids cultured in either HM-N2B27 or NDiff227, with each experimental replicate denoted by a different colour. Statistical significance was performed on the mean roundness, as described in Fig. 2A. Data representative of three biological replicates, with each replicate condition containing between 30 and 70 individual gastruloids.

Development • Supplementary information

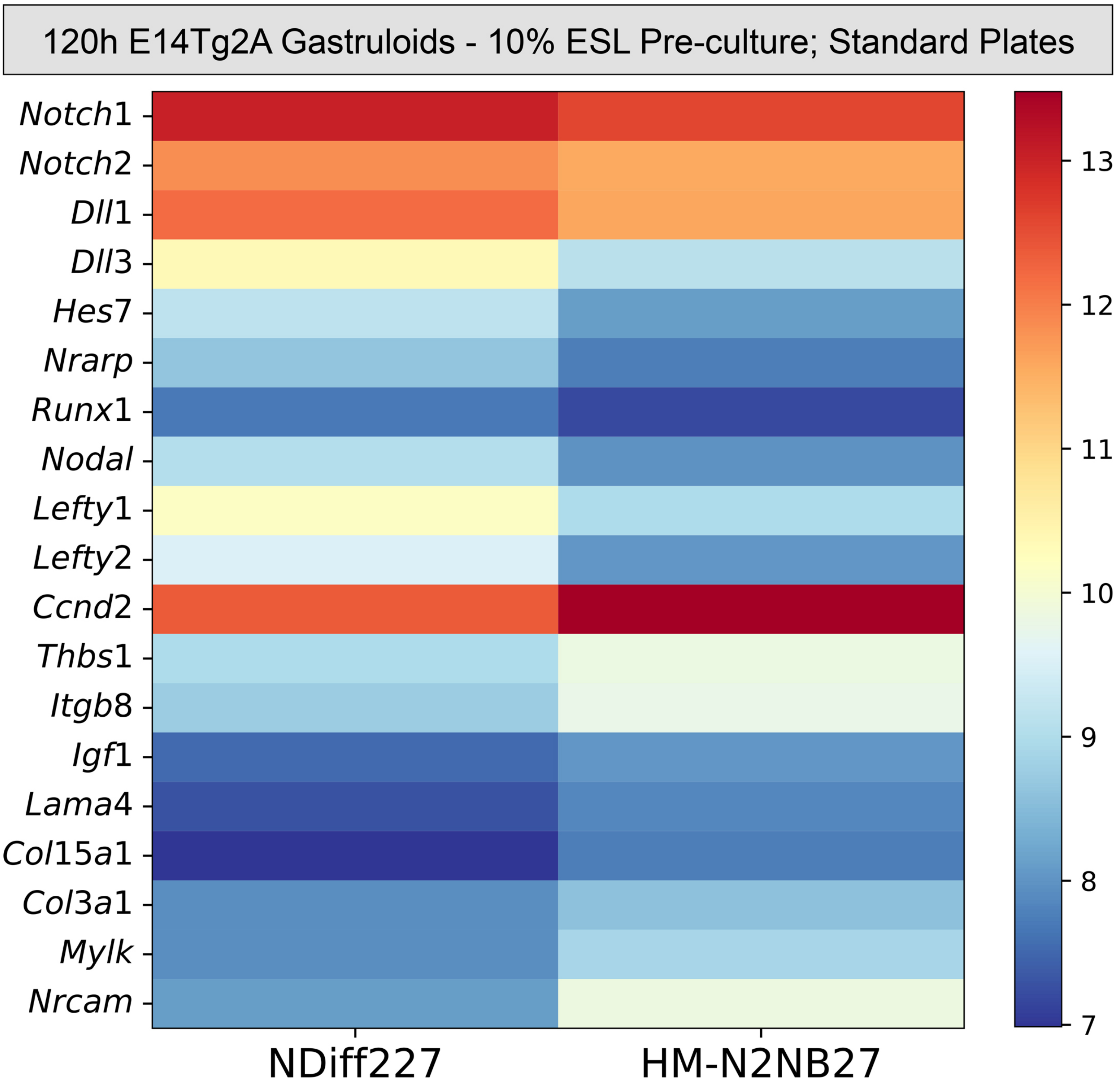

Fig. S5. Specific transcriptomic differences between HM-N2B27 and NDiff227 gastruloids at 120h. Heatmap (mean of the biological replicates variance-stabilised read counts) highlighting selected genes related to the Nodal, Notch and PI3K/Akt signalling pathways that were differently expressed between the two media conditions. Also shown is the expression of selected genes related to biological processes differently enriched in HM-N2B27 and NDiff227 gastruloids (e.g. *Col3a1*, *Mylk*, *Nrcam*; **Fig. 4** and **Table S5**).

Development • Supplementary information

Table S1. Statistical output of the maximum mixed effects model. Tables include individual fixed effects variables, interactions and random effects for area (left), length (middle), and Elongation Index (right). Variables that were $p<0.05$ were dropped in refined models used for generating predictive data in **Fig 1C.**

Fixed Effects	Estimate	SE	*t*	*p*
Intercept	0.213	0.070	3.053	
Medium	-0.181	0.090	-2.024	0.161
Elongation	-0.170	0.031	-5.498	$<2.2e^{-16}$
Length	0.251	0.080	3.137	$<2.2e^{-16}$
Elongation: Medium	0.107	0.040	2.678	$4.783e^{-05}$
Length: Medium	0.132	0.117	1.128	0.001
Elongation: Length	0.099	0.034	2.885	0.025
Elongation: Length:Medium	-0.093	0.051	-1.828	0.068

Random Effects	Variance	SD
Replicate (Intercept)	$5.997e^{-06}$	0.002
Residual	$1.112e^{-04}$	0.011

Fixed Effects	Estimate	SE	*t*	*p*
Intercept	0.046	0.137	0.334	
Medium	0.080	0.177	0.453	$2.516e^{-05}$
Elongation	0.172	0.060	2.877	$<2.2e^{-16}$
Area	1.744	0.524	3.330	$<2.2e^{-16}$
Elongation: Medium	-0.077	0.077	-1.002	0.416
Area: Medium	-0.332	0.861	-0.386	0.001
Elongation: Area	0.029	0.228	0.129	0.443
Elongation: Area:Medium	0.303	0.374	0.812	0.417

Random Effects	Variance	SD
Replicate (Intercept)	$2.285e^{-05}$	0.005
Residual	$5.104e^{-04}$	0.023

Fixed Effects	Estimate	SE	*t*	*p*
Intercept	1.929	0.578	3.336	
Medium	0.134	0.762	0.175	$5.605e^{-05}$
Area	-13.705	2.250	-6.092	$<2.2e^{-16}$
Length	2.400	0.702	3.420	$<2.2e^{-16}$
Area: Medium	-7.942	3.797	-2.091	0.008
Length: Medium	0.558	1.021	0.546	$3.717e^{-07}$
Area: Length	7.213	2.517	2.866	$2.881e^{-05}$
Area:Length: Medium	7.428	4.999	1.486	0.137

Random Effects	Variance	SD
Replicate (Intercept)	$2.624e^{-16}$	$1.62e^{-08}$
Residual	$1.438e^{-02}$	0.012

Table S2. Number of gastruloids pooled per time-point. Numbers of gastruloids in each experimental/biological replicate for the indicated time-points, corresponding to **Fig. 1E**. Average cell numbers from three experimental replicates ± standard deviation is shown rounded to the nearest whole number. A Two Way ANOVA followed by Bonferroni's Post Hoc test was performed with the *p* value of the pairwise comparisons between N2B27 and NDiff227 at each time-point given to 4 decimal places. Only selected comparisons are shown for clarity.

Time Point	No. of Gastruloids	Average Number of cells/gastruloid	*p* value
48h	96	NDiff227: 1960 ± 65<br>HM-N2B27: 2501 ± 182	$p = 0.1811$ (N.S)
72h	48	NDiff227: 3397 ± 304<br>HM-N2B27: 5240 ± 312	$p < 0.001$ (***)
96h	24	NDiff227: 6268 ± 255<br>HM-N2B27: 11256 ± 60	$p < 0.001$ (***)
120h	12	NDiff227: 10810 ± 273<br>HM-N2B27: 21839 ± 311	$p < 0.001$ (***)

Table S3. Differentially Expressed Genes shown in the volcano plot (**Fig. 4A**).

Available for download at
https://journals.biologists.com/dev/article-lookup/doi/10.1242/dev.204774#supplementary-data

Table S4. Top 100 genes based on loadings of PCA dimensions 1 and 2 (**Fig. 4B**).

Available for download at
https://journals.biologists.com/dev/article-lookup/doi/10.1242/dev.204774#supplementary-data

Table S5. GO term analysis (**Fig. 4F,G**).

Available for download at
https://journals.biologists.com/dev/article-lookup/doi/10.1242/dev.204774#supplementary-data

Table S6. Recommendations and guidelines for reproducible generation of mouse gastruloids using NDiff®227or homemade N2B27 media

Category	Parameter	Notes
NDiff®227	Storage conditions	Store at -20 °C or, ideally, at -70 °C. Use within the validity date. Protect from light.
	Media preparation	The media should ideally be thawed one day before the experiment.
		After equilibrating at room temperature for 1 hour, place the NDiff227 bottle in a water bath at 37 ºC and shake vigorously every 10 minutes (ensure that all media inside the bottle is fully immersed). Once thawed (always protect from light), store at 4 °C for up to 2 weeks.
		Before use, allow the media to equilibrate at room temperature for 1 hour and gently shake the media to ensure thorough mixing (avoid foam formation). Always protect from light exposure.
	Quality control	NDiff227 bottles should arrive frozen, with plenty of dry ice covering them from top to bottom. Freeze-thaw cycles significantly reduce media quality.
		Media that appears yellow and/or contains dark pink layers is of poor quality. The media should display a uniform orange-red colour.
		Keep track of batch variations and always batch-test for the classical gastruloid morphology at 120 h. Keep a record of inter- and intra-batch variations, using morphometrics. The media is of poor quality if more than 30% of the gastruloids do not elongate.
Homemade N2B27	Storage conditions	Store supplements (N2 and B27) and Glutamine in individual aliquots at -70 ºC and thaw to 4ºC the day before preparing the medium. Always protect from light.
	Media preparation	Prepare individual 50mL aliquots of N2B27.
		The two media components (N2 and B27) should be prepared separately and mixed before adding the ß-mercaptoethanol. Then, gently shake to ensure thorough mixing (avoid foam formation) and store at 4ºC. All reagents should be kept on ice and protected from light.
		Before use, allow the media to equilibrate at room temperature for 1 hour and gently shake the media to ensure thorough mixing (avoid foam formation). Always protect from light exposure.
	Quality control	After mixing, the media should display a

Development • Supplementary information

		uniform orange-red colour. Remember that the media colour changes according to the CO_2 levels and pH of the solution.
		Keep accurate and detailed records of Lot Numbers and expiry dates of all media components, and always batch-test for the classical gastruloid morphology at 120 h. Keep a record of inter- and intra-batch variations, using morphometrics. The media is of poor quality if more than 30% of the gastruloids do not elongate.
		If new batches of complete medium components are required, substitute them individually and examine how they make gastruloids before changing all component batches.
Stem cell culture and gastruloid development	2D culture conditions	Cells should be in culture for at least 3 passages before the gastruloid protocol.
		Each cell line will require optimisation for the number of seeded cells and the days between each passage. Ensure a confluence of 70 to 80% before making gastruloids or freezing the cells.
		Ensure cell counts on passage days are consistent within each media batch; a reduction in cell count over time will give suboptimal gastruloids.
		Excessive incubation with Trypsin, TrypLE or similar reagents has a detrimental effect on gastruloid formation.
		Test various serum suppliers. Always batch-test and keep records (do this for all reagents).
		Test frequently for Mycoplasma.
		Avoid using antibiotics, as they may mask underlying contamination or infection.
	Gastruloids	Avoid preparing and changing the media on an excessive number of gastruloid plates simultaneously. Gastruloids are negatively affected by long durations out of the incubator during preparation and media changes.
		The first day of the protocol is critical and should last a maximum of 30 to 45 minutes.
		Optimal gastruloid formation requires cell viability >95%.
		Avoid the formation of bubbles/foam in all steps of the gastruloid protocol.
		On the first day of the protocol, wash twice the single cell suspension with PBS. This ensures complete removal of the dissociating reagent and results in higher-quality gastruloids.
		Each cell line will require optimisation for the number of seeded cells; plate a range of cell

Development • Supplementary information

		numbers (e.g. 150 – 400 cells/well). If gastruloids display a high percentage of multi-axes, reduce the initial cell number.
		Ensure cell count accuracy and pipetting precision.
		Always batch-test new reagents, avoid freeze-thawing (use individual aliquots) and follow the manufacturer's storage and validity information.
		Use non-adherent U-bottomed 96-well plates to allow cells to aggregate naturally. Ultra-low attachment plates can mask the capacity of cells to make gastruloids and result in lower quality aggregates.
		Fill the outer wells of the 96-well plate with 150 uL of PBS to minimise the evaporation that normally occurs there and affects gastruloid development.
		Every addition of N2B27 should be administered vigorously to ensure agitation and limit the attachment of gastruloids to the well surface.
		Avoid using antibiotics, as they may mask underlying contamination or infection. Discard all gastruloids with any sign of bacterial or fungal infection.
		Wash gastruloid at the end of the protocol, take pictures and assess their morphology and the location/expression of key axial markers (e.g. Brachyury). Keep records of all experiments.
